# The VP1/2 Protein of a New Recombinant PRV Strain Promotes the Infectivity and Pathogenicity of PRV in Northeastern China

**DOI:** 10.1155/2024/1575103

**Published:** 2024-02-17

**Authors:** Yan Pan, Xin Yao, Tian-Ning Yang, Jin-Long Li, Dong-Fang Shi

**Affiliations:** ^1^College of Veterinary Medicine, Northeast Agricultural University, Harbin 150030, China; ^2^Key Laboratory of the Provincial Education Department of Heilongjiang for Common Animal Disease Prevention and Treatment, Northeast Agricultural University, Harbin 150030, China; ^3^Heilongjiang Key Laboratory for Laboratory Animals and Comparative Medicine, Northeast Agricultural University, Harbin 150030, China

## Abstract

Pseudorabies virus (PRV) is an acute infectious disease characterized by neurological and respiratory symptoms. In order to have a better understanding of the current prevalence of PRV in northeastern China, a strain of PRV was isolated by us. Then, protein structure analysis and pathogenicity testing of the virus were performed to give insight into the characterization of the isolated PRV strains. In this study, the PRV strain named CH/HLJPRVJ/2023 was isolated and identified. Genome-wide phylogenetic analysis shows CH/HLJPRVJ/2023 and HeN1 have higher homology. The CH/HLJPRVJ/2023 strain had the highest homology with HeN1 strain (97.3%) and the lowest homology with Bartha-K61 (89.2%). Recombinant evolution analysis shows CH/HLJPRVJ/2023 shows many variants in OBP, AN, UL21, UL17, VP11/12, and VP1/2 fragments, which predict its unique genetically. VP1/2, an effector protein of capsid transport and neuroinvasion, has mutations and deletions in its amino acids, which cause changes in the protein conformation of CH/HLJPRVJ/2023. Besides the typical neurologic and respiratory lesions, infection with highly pathogenic CH/HLJPRVJ/2023 can lead to damage to the colonic villi and colonic barrier in piglets. This study will provide a basis for knowledge about the prevalence, genetic evolution, and vaccine optimization of endemic PRV strains in northeastern China.

## 1. Introduction

Pseudorabies virus (PRV), primarily infecting pigs, has caused extensive disease spread and significant economic losses worldwide since its discovery [1]. Infected pigs typically exhibit symptoms such as slow growth, respiratory system disorders, and reproductive failure [2]. PRV viral particles are spherical with a diameter of 150–180 nm. PRV consists of an envelope enclosing a capsid with associated tegument proteins [3]. While the structural features of PRV particles are generally consistent, however, the virulence of different PRV strains can be influenced by variations in the virus genome [4].

In recent years, mutated strains of PRV have garnered widespread attention worldwide. China effectively controlled the spread of pseudorabies through the Bartha-K61 vaccine [5]. However, vaccine administration has sometimes failed to prevent large-scale infections since 2011 [6]. Genetic analysis of strains has indicated that mutations in the PRV genome may be the primary reason behind this result [7]. Human-derived PRV strain hSD-2019/1 has been isolated from cerebrospinal fluid, causing neurological symptoms [2, 8]. Research also highlights the significance of the VP1/2 protein in retrograde axonal transport and neural invasion by herpesviruses [9, 10]. Additionally, the VP1/2 tegument protein is considered as a prime candidate for bridging the gap between the alpha herpesvirus capsid and tegument [11]. VP1/2 protein plays a crucial role in viral genome packaging and host cell invasion during PRV infection [12]. Scientists speculate that changes in the protein sequence and conformation of VP1/2 could modify the infectivity of the PRV [13].

In this study, a recent PRV strain from northeastern China was isolated and analyzed, and genomic mutations were detected and structural changes in VP1/2 protein were identified. Furthermore, these isolated strains were used to challenge newborn piglets to assess their pathogenicity. These findings contribute to our understanding of the evolution information of PRV and could offer new insights into the immune escape capabilities of PRV strains.

## 2. Methods and Materials

### 2.1. Ethics Statement

Animal experiments on piglets were approved by the Laboratory Animal Ethics Committee of Northeast Agricultural University (NEAUEC20230383). The experiments were conducted in strict accordance with the Guidelines for the Care and Use of Laboratory Animals of the Ministry of Science and Technology of the People's Republic of China.

### 2.2. Isolation and Identification of Viruses

In 2023, the cerebellar tissues were collected from diseased piglets at pig farms in Heilongjiang province, China. These piglets had a raised temperature and clinical signs of salivation and cough before death. The collected tissues were well homogenized in PBS and centrifuged at 3,000x *g* for 20 min. The viral fluid was passed through a purification column to isolate viral DNA according to the instructions of the manufacturer (TIANGEN, China). Then, the presence of PRV was demonstrated by amplifying the gD gene with PCR (MiniAmp Plus, Thermo Fisher, America) and qPCR (Line-Gene9660, Bioer, China). The sequences of the gD primers used in this study are listed in *Supplementary 1*. Vero E6 cells were removed from liquid nitrogen and rapidly melted in water at 37°C. Cells were cultured in three generations in DMEM medium (HyClone, America) containing 10% FBS (HyClone, America). The supernatant was passed through a 0.22 *μ*m filter membrane and inoculated into a monolayer of Vero cells. Passages were performed up to 20 generations, and the supernatant from each generation was collected.

### 2.3. Transmission Electron Microscopy (TEM) Observation

When the virus was cultured to the 20^th^ generation, the stained viral fluid was observed with transmission electron microscopy (TEM; Hitachi HT7650, Japan).

### 2.4. Plasmid and Full-Length Genome Sequencing of CH/HLJPRVJ/2023 Strain

Isolated viral fluids were collected and used for whole genome sequencing performed by Guangdong Megger Genetics Technology Co. The gD fragment plasmid of CH/HLJPRVJ/2023 was deposited in our laboratory.

### 2.5. Determination of Viral Replication Rate and Viral Titer in Viral Fluid

Vero E6 cells (2 × 10^6^/mL) were inoculated into six-well cell culture plates and grown in a 5% CO_2_ incubator for 24 hr. A standard curve for the gD gene was performed using the plasmid. The viral replication rate in the viral solution of the 1^st^ and 20^th^ generations was determined by setting passage 0%(P0)–100% viral replication rate. The TCID_50_ of the virus was calculated according to the Reed–Muench method, and lgTCID_50_ was calculated as the viral titer of the 8^th^ and 20^th^ generation viral fluids.

### 2.6. Analysis of Sequence and Protein Structure

Phyre2 was used to validate the DNAMAN comparison results of the amino acid sequence of each protein. Then, Phyre2 was then used for homology modeling of protein tertiary structures (https://www.sbg.bio.ic.ac.uk/phyre2/html) along with SWISS-MODEL (https://swissmodel.expasy.org/interactive). The mutation position of the amino acid was ascertained by FirstGlance in Jmol (http://www.sbg.bio.ic.ac.uk/~phyre2/html/page.cgi?id=index).

### 2.7. Infection Experiment of Newborn Piglets

Twenty healthy newborn Duroc piglets were taken and reared in the Laboratory Animal Center of Northeast Agricultural University. They were fed daily with piglet weaning milk powder (Anyou Feed, Beijing, China) for artificial milk exchange. After 2 days of acclimatization, the piglets were randomly divided into two groups. PBS was added to the milk powder as a mock infection group (Mock), while CH/HLJPRVJ/2023 viral solution with the same titer as P20 was added to the milk powder as a CH/HLJPRVJ/2023-infected group (HLJPRVJ) as a daily diet for days 3–6.

### 2.8. Detection of Indirect Immunofluorescence

Isolated piglet jejunal tissues were fixed in 4% paraformaldehyde for 24 hr, washed in PBS, and left overnight in 30% sucrose solution. Subsequently, it was embedded with OTC frozen section embedding agent and stored frozen at −20°C in a refrigerator. Later, the sections were cut into 15 mm thin slices in a frozen sectioning machine (Leica CM1950, Germany) and transferred to slides. After rewarming at room temperature, circles were drawn with a histochemical pen and the excess embedding agent was washed away with PBS. The frozen sections or cell crawls obtained above were incubated with 10% FBS for 30 min and washed three times with PBS. Then, it incubated with PRV primary antibody (laboratory self-produced) at 4°C overnight, and set with Alexa Fluor 594 (bs-0368G-AF594, Bioss, China) fluorescent secondary antibody at room temperature for 1 hr. Subsequently, the slices were blocked with an antifluorescence quenching blocking agent containing DAPI (P0131, Beyotime, China), and were immediately visualized under the fluorescence microscope. Vero cells were inoculated in 12-well plates and left to grow to 70% monolayer. Cells were fixed with 4% paraformaldehyde and Triton X-100 for 30 min. Closure and primary antibody incubation were performed using the same steps as above and incubated with Alexa Fluor 488 fluorescent secondary antibody (bs-0295G-AF488, Bioss, China) for 1 hr at room temperature, followed by sealing of the plate with antifluorescent quenching sealer containing DAPI and immediate observation under a fluorescence microscope (Leica DMi8, Germany).

### 2.9. Preparation and Staining of Tissue Sections

The isolated piglet brain, lung, and colon tissues were fixed in 4% paraformaldehyde for 24 hr, followed by gradient dehydration, de-ethanolization, de-xylene, and then transferred to pure paraffin in an oven. Embedding was performed using a paraffin embedding machine and fixed with an embedding box. After the paraffin was completely solidified, sections were made with a fully automatic rotary slicer, followed by gradient rehydration, and stained with the hematoxylin eosin (H.E) method. After gradient dehydration and ethanol, the slides were moistened with xylene, sealed with a clear resin, and observed under a light microscope (Leica DM300, Germany).

### 2.10. Scanning Electron Microscope (SEM) Observation

Isolated piglet colon tissue was cut into 3-mm^3^ pieces and fixed in 2.5% glutaraldehyde for 24 hr. Colon samples were progressively dehydrated with gradient ethanol and then de-ethanolized with tert-butanol. Samples placed in pure tert-butanol appeared to crystallize after the colon samples were refrigerated at 4°C overnight. After freeze-drying, the samples were sprayed with gold treatment and subsequently observed under a scanning electron microscope (SEM; Hitachi SU8010, Japan).

### 2.11. Statistical Analysis

Statistical comparisons were performed with GraphPad Prism software (version 8.3). Error bars indicate standard error (±SE). Data were analyzed by student's *t*-test. *P* < 0.05 were considered statistically significant.  ^*∗*^*P* < 0.05,  ^*∗∗*^*P* < 0.01, and  ^*∗∗∗*^*P* < 0.001 were considered statistically significant.

## 3. Result

### 3.1. Isolation, Identification, and Culture of CH/HLJPRVJ/2023

**Figure 1:**
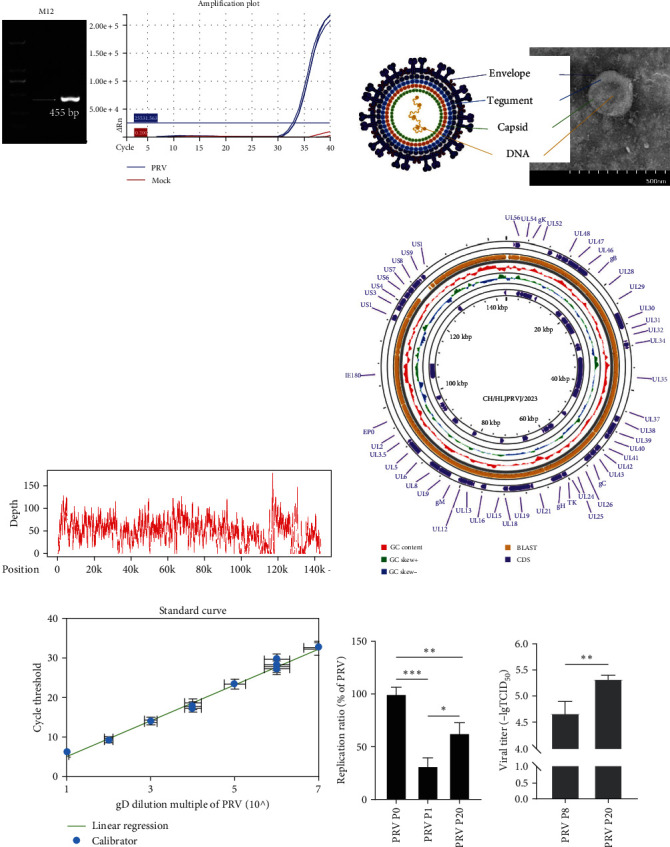
Isolation, identification, and culture of CH/HLJPRVJ/2023. (a) Detect PRV positive results in the cerebellar, controls were negative. (b) The supernatant of the 20^th^ passage of infected Vero cells was collected for transmission electron microscopy (TEM), and the bilayer structure of PRV particles was observed. (c) In-depth results of viral genome sequencing. (d) CH/HLJPRVJ/2023 genome map, including GC content, CDS region, and blast to Bartha-K61. (e) gD gene cloning plasmid was constructed to prepare PRV standard curve (*n* = 3). (f) Organization grinding fluid as P0 generation, to use its infection Vero cell on the freeze–thaw centrifugal clear for P1, in turn, get to P20 generation, detecting the three generations of copy number of the virus (*n* = 3,  ^*∗*^*P* < 0.05,  ^*∗∗*^*P* < 0.01, and  ^*∗∗∗*^*P* < 0.001). CH/HLJPRVJ/2023 was blindly passaged to P8 generation to develop a cytopathic effect, and the TCID_50_ of P8 and P20 generations was detected (*n* = 3,  ^*∗∗*^*P* < 0.01).

To detect the antigen collected in pig farms in Heilongjiang, China, total DNA was extracted from cerebellar and separated. And PCR and qPCR results showed that the samples were tested positive for PRV (Figure 1(a)). Then, TEM was performed to confirm only PRV viral infection. When the virus was cultured to the 20^th^ generation, the viral fluid was observed with TEM. There is only one form of virus particle in all fields of view. And 180 nm-sized PRV viral particles, including glycoprotein fibrils on the surface of vesicle membranes, could be clearly observed (Figure 1(b)). The virus was named CH/HLJPRVJ/2023 strain by us. Then, we performed full-length genome sequencing analysis of this strain. Sequencing results showed that the depth of sequencing was generally consistent and matched the results of single virus infection (Figure 1(c)). The full length of the genome of the CH/HLJPRVJ/2023 strain was 143,219 bp. The gene alignment of the CH/HLJPRVJ/2023 strain and differences from Bartha-K61 in BLAST are shown in Figure 1(d). To quantify viral replication power, we constructed the recombination plasmid targeting the gD plasmid of PRV. And then the standard curves were plotted (Figure 1(e)). The viral replication rate was determined for P0, P1, and P20 cells. A significant decrease in viral replication was observed for P1 and P20. Compared to P1, there was a significant rebound in the viral replication rate of P20. The TCID_50_ was determined by the Reed–Muench method. And then the viral titers were determined for Vero E6 cells of 8^th^ generation and 20^th^ generation, respectively. The viral titer was 4.67 TCID_50_/mL for P8 and 5.33 TCID_50_/mL for P20. There was a significant increase in the viral titer for P20 compared with P8 (Figure 1(f)).

### 3.2. Full Gene Phylogenetic Analysis of CH/HLJPRVJ/2023

**Figure 2:**
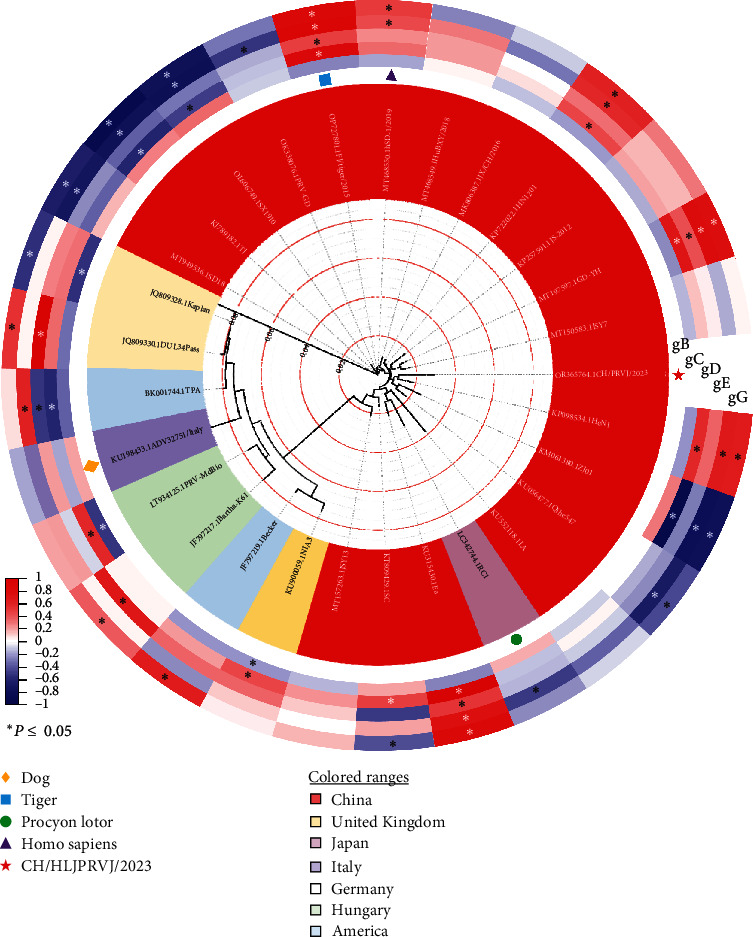
CH/HLJPRVJ/2023 genome-wide phylogenetic tree analysis. Downloaded from NCBI databases random 28 strains of PRV genome reference sequence analysis, PRV in China, Japan, Britain, the United States, Italy, Germany, and Hungary, mainly popular in China. And the hosts of reference strains included tiger, *Procyon lotor*, and homo sapiens in addition to *Sus scrofa*. The differential heat map was drawn according to the correlation analysis between the reference strain and the isolates gB, gC, gD, gE, and gG proteins ( ^*∗*^*P* < 0.05).

To further investigate the evolutionary relationship between the CH/HLJPRVJ/2023 strain and other PRV strains, a phylogenetic analysis based on the entire genomic sequences of the CH/HLJPRVJ/2023 strain and other strains (*Supplementary 1*) available in GenBank was performed. In Figure 2, the phylogenetic tree of PRV complete genomes exhibited the same branch from the HeN1 strain in the tree generated. Moreover, the CH/HLJPRVJ/2023 strain shares higher similarity with some strains found in China. The gB, gC, gD, gE, and gG proteins of CH/HLJPRVJ/2023 strain showed a significant correlation (*P* < 0.05) with those of other strains (Figure 2). CH/HLJPRVJ/2023 strain and GD-YH, FJ/tiger/2015, and Ea strains showed significant positive correlations in the fragments of gC, gD, gE, and gG (*P* < 0.05). *Supplementary 2* showed the homology analysis of the CH/HLJPRVJ/2023 strain and other PRV strains. The CH/HLJPRVJ/2023 strain had the highest homology with HeN1 strain (97.3%), and the lowest homology with Bartha-K61 (89.2%; *Supplementary 2*).

### 3.3. Amino Acid Mutations of CH/HLJPRVJ/2023

**Figure 3:**
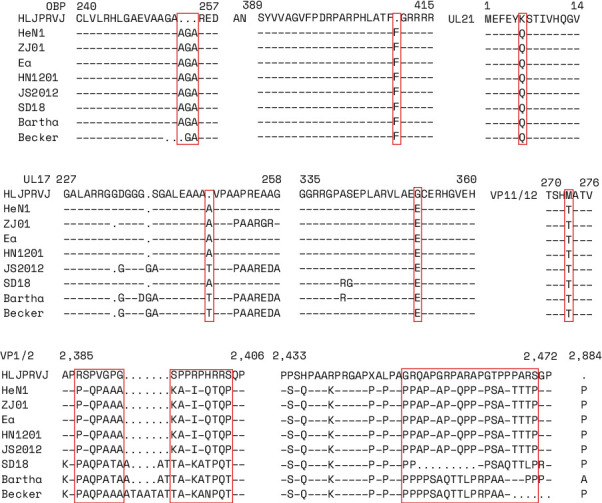
According to the analysis of the evolutionary tree selection and isolates the recent HeN1 strain, other subtypes of Becker and Bartha, the same subtype of different branch SD18, HN1201, JS2012, ZJ01, and Ea strain as representatives of sequence alignment. (a) OBP protein to contrast. (b) AN protein to contrast. (c) UL21 protein to contrast. (d) UL17 protein to contrast. (e) VP11/12 protein to contrast. (f) VP1/2 protein to contrast. The red box represents the unique evolutionary characteristics of CH/HLJPRVJ/2023.

To determine the specificity of CH/HLJPRVJ/2023, DNAMAN was used to compare and analyze the amino acids of eight randomly selected strains. The results showed that three amino acids were deleted in OBP protein (254 aa; Figure 3(a)). And there is one amino acid deletion in both AN protein (410 aa) and UL17 protein (248 aa; Figures 3(b) and 3(d)). Three amino acid mutations, K-Q (6 aa), G-E (352 aa), and M-T (273 aa), resulted from in the UL21, UL17, and VP11/12 proteins, respectively (Figure 3(c)–3(e)). Consecutive amino acid mutations and deletions were found in VP1/2 (2387–2406 aa and 2451–2470 aa; Figure 3(f)). The results also showed that there were certain amino acid-specific conserved among the strains, and the conservation of these regions was the basis for identifying PRV strains. These results can provide a basis for the identification of PRV strains.

### 3.4. Mutations and Deletions of Amino Acids Altered AP1/2 Protein Spatial Structure of CH/HLJPRVJ/2023

**Figure 4:**
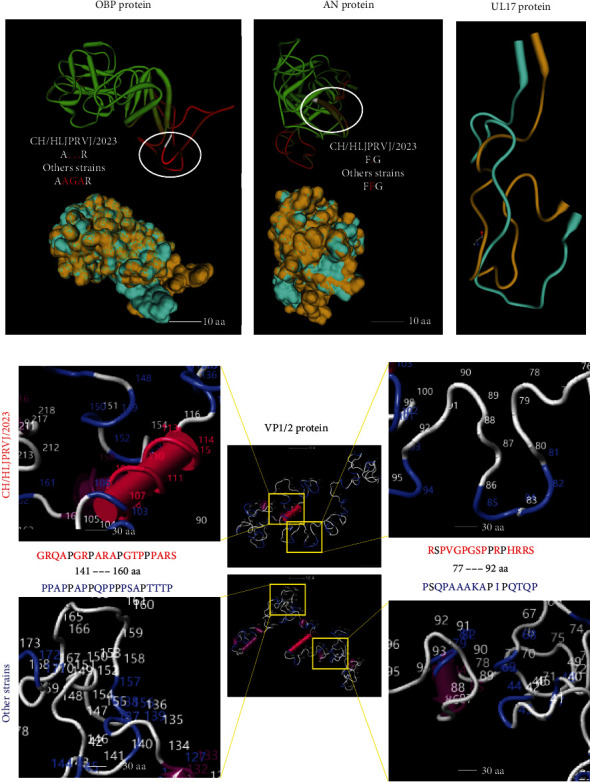
According to the differences in amino acid protein three-dimensional structure comparison. (a) For OBP protein (UL9) 3D model alignment, the deletion of three amino acids of AGA at 254–255 aa resulted in subsequent helical alterations in the isolates compared with other strains. (b) For the 3D model alignment of the AN protein (UL12), the deletion of phenylalanine at 410–411 aa in the isolates resulted in the production of specific bumps in the protein compared with other strains. (c) Comparison of the 3D model of UL17 protein showed that the deletion of alanine at 248–249 aa shifted but did not change the structure of the isolate compared with other strains. (d) 2,310–2,530 aa range of the VP1/2 protein (UL36) was simulated, and the protein tertiary structure of the isolates was found to have lost an alpha helix at 77–92 aa and added an alpha helix at 141–160 aa compared to other strains. In addition, four additional alpha helices were lost within this simulation range due to generalized mutations in the isolates.

One of the analyzed strains was selected to compare protein spatial structure modeling, and 3D models of proteins that appeared to have amino acid mutations and deletions were constructed to explore changes in protein structure and function. For OBP protein (UL9) 3D model alignment, the deletion of three amino acids of AGA at 254–255 aa resulted in subsequent helical alterations in the isolates compared with other strains (Figure 4(a)). In AN protein (UL12), the deletion of phenylalanine at 410–411 aa in the isolates resulted in specific bumps in the protein compared with other strains (Figure 4(b)). Comparison of the 3D model of UL17 protein showed that the deletion of alanine at 248–249 aa shifted but did not change the structure of the isolate compared with other strains (Figure 4(c)). Then, the 2,310–2,530 aa of the VP1/2 protein (UL36) was simulated, and the protein tertiary structure of the isolates was found to have lost an alpha helix at 77–92 aa and added an alpha helix at 141–160 aa compared to other strains (Figure 4(d)). In addition, four additional alpha helices were lost within this simulation range due to generalized mutations in the isolates.

### 3.5. Recombination Analysis of CH/HLJPRVJ/2023

**Figure 5:**
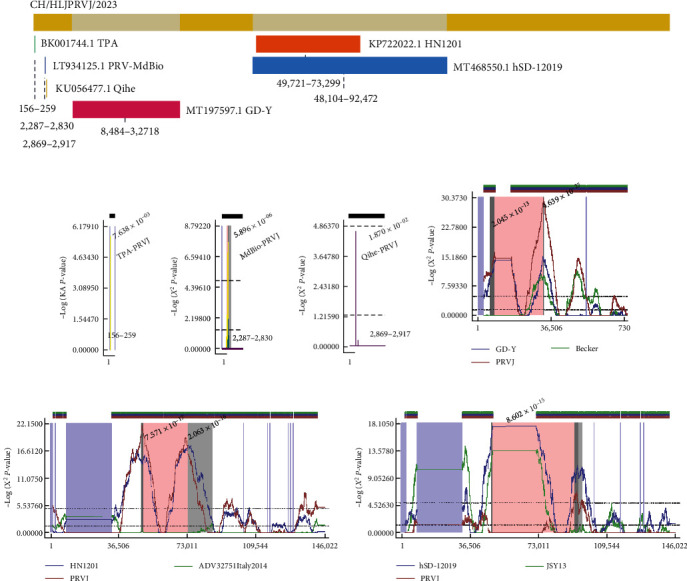
A total of 29 isolates and reference strains were subjected to recombination analysis by RDP4. (a) CH/HLJPRVJ/2023 with TPA, PRV-MdBio, Qihe, GD-Y, HN1201, and hSD-12019 restructuring information overview. (b) The possibility of recombination of TPA with isolates was analyzed by GENECONV. (c) The possibility of recombination of PRV-MdBio with isolates was analyzed by MaxChi. (d) The possibility of recombination of Qihe with isolates was analyzed by MaxChi. (e) The possibility of recombination of GD-Y with isolates was analyzed by Chimaera. (f) The possibility of recombination of HN1201 with isolates was analyzed by Chimaera. (g) The possibility of recombination of hSD-12019 with isolates was analyzed by Chimaera. RDP, Chimaera, BootScan, 3seq, GENECONV, MaxChi, and SiScan in more than three significant are considered valid.

To determine if potential recombination events occurred in the evolution of CH/HLJPRVJ/2023 strain, recombination analyses were performed on the strain along with reference strains retrieved from GenBank using the RDP4 software (Figure 5(a)). The recombination probability of the strains was predicted using RDP, Chimaera, BootScan, 3seq, GENECONV, MaxChi, and SiScan algorithms, respectively. The results showed that the CH/HLJPRVJ/2023 strain is a recombination of the TPA (GenBank number BK001744.1), PRV-MdBio (GenBank No. LT934125.1), Qihe547 (GenBank No. KU056477.1), GD-YH (GenBank No. MT197597.1), HN1201 (GenBank No. KP722022.1), and hSD-1/2019 (GenBank No. MT468550.1) strains obtained by homologous recombination (Figure 5(b)–5(g)). All above recombination events had at least three types of algorithms showing that the recombination events were plausible (*P* < 0.05; *Supplementary 1*).

### 3.6. Pathologic Analysis of Newborn Piglets with Highly Pathogenic CH/HLJPRVJ/2023

**Figure 6:**
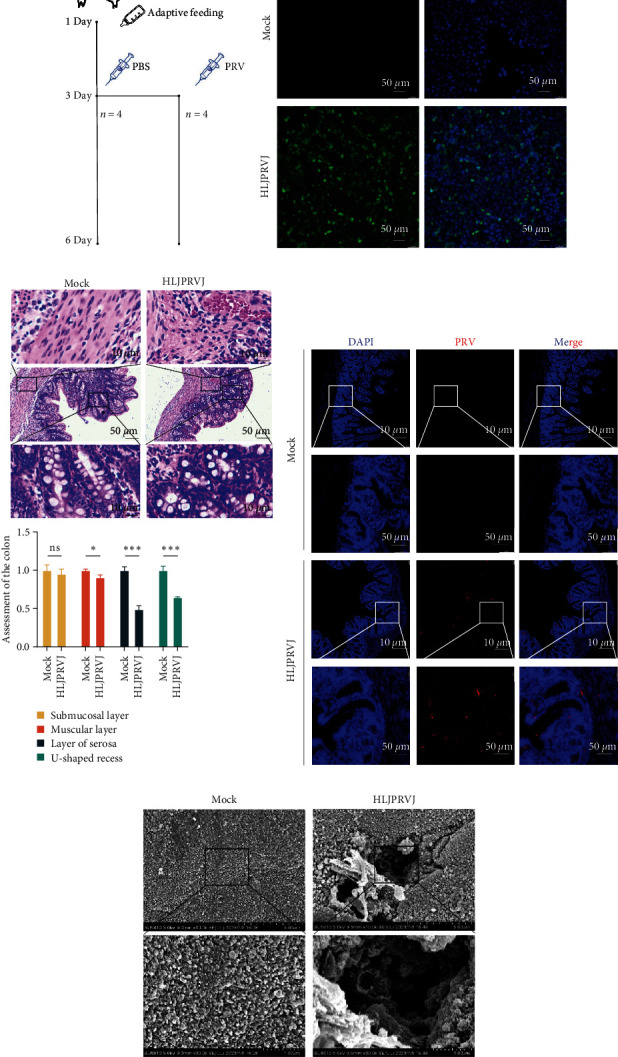
Pathological analysis of newborn piglets infected with highly pathogenic CH/HLJPRVJ/2023. (a) Newborn piglets were orally infected with PRV (3 dpi) after 48 hr artificial milk replacement, and the mock infection group was treated with PBS instead (*n* = 4). (b) CH/HLJPRVJ/2023 P20 was used to infect animals, and Vero was infected before experiments for immunofluorescence identification. (c) Colonic staining results were quantified using the mock infection group as a control (*n* = 3,  ^*∗*^*P* < 0.05, and  ^*∗∗∗*^*P* < 0.001). (d) The animal model of infection was established by immunofluorescence of the jejunum. (e) Results of scanning electron microscopy of the colon.

To validate the highly pathogenic CH/HLJPRVJ/2023, we performed infection experiments in piglets. Newborn piglets were orally infected with PRV (3 dpi) after 48 hr artificial milk replacement, and the Mock group was treated with PBS instead (Figure 6(a)). The piglets of CH/HLJPRVJ/2023 showed some cough symptoms, but did not cause death. Then, an effective PRV antibody (Figure 6(b)) was used to detect the existence of PRV in the jejunum. There was no red fluorescence present in the Mock group, while there was bright red fluorescence in the jejunal villi of the HLJPRVJ group, which represented the existence of PRV (Figure 6(d)). Alveolar epithelial cell hyperplasia was observed in the HLJPRVJ group and was accompanied by inflammatory cell infiltration. Mononuclear cell infiltration and localized small vascular endothelial cell swelling were found in the brain of the HLJPRVJ group (*Supplementary 2*). The muscular layer cells were swollen, and the length of the villi was shortened in the colon. Compared with the Mock group, the HLJPRV group showed increased pathology in the muscular layer and layer of serosa, and the depth of the U-shaped recess of the villi was reduced (*P* < 0.05 and *P* < 0.001; Figure 6(c)). TEM results showed that the Mock group showed tidy and dense microvilli morphology. Whereas, holes appeared in the HLJPRV group, which predicted the disruption of microvilli connections in the colon (Figure 6(e)).

## 4. Discussion

PRV has been prevalent in Chinese pig farms for nearly 7 decades and caused major economic losses to the pig industry. Despite proactive control measures, many pig farms still experience intermittent and collective outbreaks of PRV [6]. Even though pigs have been vaccinated with the Bartha-K61 vaccine, the mortality rate among infected piglets in China remains as high as 50% [14]. In recent years, due to viral mutations, vaccines derived from classical strains have proven insufficient to protect against currently prevalent strains [15, 16]. Therefore, it is of great significance to monitor and evolution analysis for the prevention and control of pseudorabies, and it can provide key information for the development and application of PRV vaccines.

This study detected PRV in diseased pigs. Molecular biology analysis indicates that the CH/HLJPRVJ/2023 strain from Heilongjiang province, China, represents a PRV strain. The recent HeN1 strain, other subtypes of Becker and Bartha, the same subtype of different branches SD18, HN1201, JS2012, ZJ01, and Ea strains are regarded as representatives of sequence alignment [17]. And there are notable genetic variations between classical prevalent PRV strains and the CH/HLJPRVJ/2023 strain. Increasingly, these strains are being discovered and gaining attention [7]. The currently found PRV variants not only differ in pathogenicity from classical strains, but also differentiate among different hosts [18]. Therefore, it is necessary to learn more about the diversity of PRV to enhance our understanding of their biological characteristics and evolution.

In contrast to traditional vaccine strains, CH/HLJPRVJ/2023 exhibits differences in pathogenicity and genomic sequences compared to classical PRV strains in China and abroad. CH/HLJPRVJ/2023 shares a close genetic relationship with the HeN1 strain. And Ea and Bartha-K61 strains are significantly different from this new strain. The mutation or deletion of amino acid sites in the CH/HLJPRVJ/2023 strain differs from all other strains, which may have a positive impact on the prevalence of PRV [2, 19]. Predicted genetic recombination events suggest credible homologous recombination in CH/HLJPRVJ/2023, GD-Y, HN1201, and hSD-12019. Similar to the results of previous studies, this could indicate the existence of interrelated mutations among various PRV strains [20, 21]. Moreover, CH/HLJPRVJ/2023 possibly mutated in other strains. Practically, it could mean that the Bartha-K61 vaccine does not provide potent protection [22]. Genetic changes in CH/HLJPRVJ/2023 could be a significant factor in immune evasion.

VP1/2 is noted for its deubiquitinase activity resulting from a cysteine protease domain in the *N* terminus [13]. The VP1/2 protein binds to the capsid. In general, the basic structural domains in VP1/2 are relatively stable and conserved to maintain the stability of the viral structure [11, 23]. Changes in the distribution of amino acids and protein conformation of VP1/2 could result from adaptive changes in the virus within its host. Multiple regions of VP1/2 contribute to the interaction, and a large proline-rich sequence located in half of the protein was identified as one determinant [9, 13]. @Regions rich in glycine and arginine could contribute to conformational changes, indirectly affecting the virus's infectivity [12].

After conducting infection experiments on newborn piglets, we observed that CH/HLJPRVJ/2023 exhibits strong pathogenicity. PRV infection leads to damage to the colonic villi and colonic barrier in piglets. Due to the high pathogenicity of CH/HLJPRVJ/2023, we suspect that changes in PRV strain protein conformation may be due to mutations at amino acid positions, which could affect its replication, transmission, and immune evasion [24, 25]. These changes may result in overall immune failure in pig populations, as our research results indicate. Additionally, the constant evolution of PRV strains poses a significant challenge to our detection techniques [26].

## 5. Conclusion

!In conclusion, this study reports the discovery of an epidemic strain of PRV in 2023 from Heilongjiang province, China, and it is named the CH/HLJPRVJ/2023 strain. Genome-wide phylogenetic analysis shows CH/HLJPRVJ/2023 and HeN1 have higher homology (97.3%). Compared with other strains, CH/HLJPRVJ/2023 shows many variants in OBP, AN, UL21, UL17, VP11/12, and VP1/2 fragments, which predict its unique genetically. VP1/2, an effector protein of capsid transport and neuroinvasion, has mutations and deletions in its amino acids, which cause changes in the protein conformation of CH/HLJPRVJ/2023. Besides the typical neurologic and respiratory lesions, infection with highly pathogenic CH/HLJPRVJ/2023 can lead to damage to the colonic villi and colonic barrier in piglets. This study will provide a basis for knowledge about the prevalence, genetic evolution, and vaccine optimization of endemic PRV strains in northeastern China.

## Data Availability

All data used to support the findings of this study are available from the corresponding author upon request.

## Supplementary Materials

Supplementary 1 Table S1: PRV strains used in this study. Table S2: PRV strains used in this study. Table S3: analysis of CH/HLJPRVJ/2023 recombinant evolution.

Supplementary 2 Figure S1: homology analysis of the CH/HLJPRVJ/2023 strain and other PRV strains. Figure S2: typical damage in the brains and lungs.
