# Supplementary material for: The VP1/2 Protein of a New Recombinant PRV Strain Promotes the Infectivity and Pathogenicity of PRV in Northeastern China
Source: Transbound Emerg Dis. 2024 Feb 17;2024:1575103. doi: 10.1155/2024/1575103 (PMC12020405; doi:10.1155/2024/1575103)
Supplement: Supplementary 1 — Table S1: PRV strains used in this study. Table S2: PRV strains used in this study. Table S3: analysis of CH/HLJPRVJ/2023 recombinant evolution. [file 1575103.f1.docx]

**Supplementary Figure 1.** **Homology analysis of** **the CH/HLJPRVJ/2023 strain and other PRV strains.**


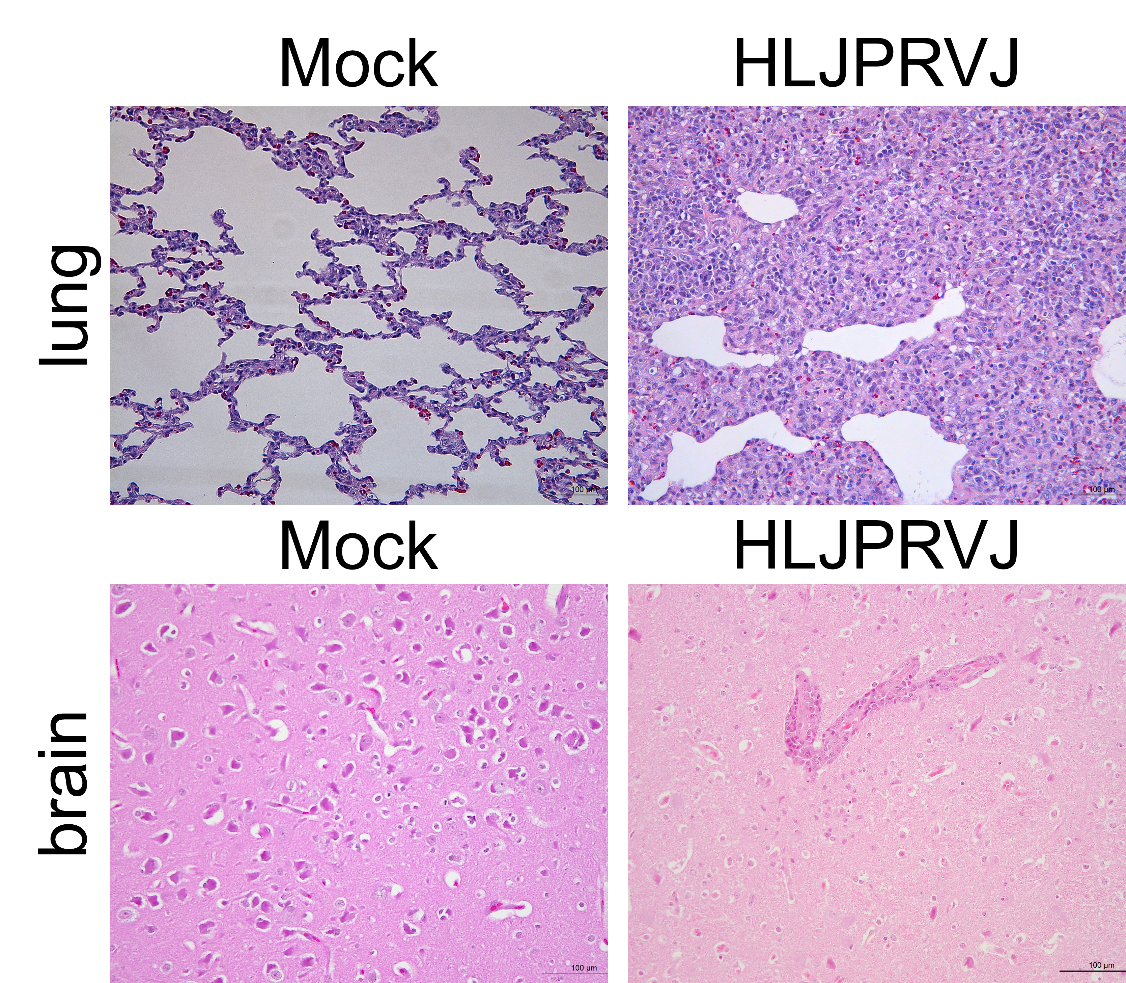


**Supplementary Figure 2. Typical damage in the brains and lungs.**
